# The Relation between Periodontopathogenic Bacterial Levels and Resistin in the Saliva of Obese Type 2 Diabetic Patients

**DOI:** 10.1155/2017/2643079

**Published:** 2017-08-23

**Authors:** Natheer Al-Rawi, Farah Al-Marzooq

**Affiliations:** ^1^College of Dental Medicine, University of Sharjah, Sharjah, UAE; ^2^Sharjah Institute for Medical Research, University of Sharjah, Sharjah, UAE

## Abstract

This study aims to investigate the relation between resistin and periodontopathogenic bacterial levels in the saliva of obese adults compared to healthy control and to examine whether salivary resistin can serve as a biomarker of type 2 diabetes in obese patients. A total of 78 saliva samples were collected from patients attending to the University Dental Hospital, Sharjah, UAE. The patients were divided into three equal groups: obese diabetics, obese nondiabetics, and nonobese nondiabetic control. Salivary resistin was measured using ELISA. The levels of bacterial species associated with periodontitis (*Treponema denticola*, *Porphyromonas gingivalis*, *Tannerella forsythia*, and *Actinobacillus actinomycetemcomitans*) and gingivitis (*Fusobacterium* spp.) were measured using real-time PCR. Both salivary resistin and periodontopathogenic bacteria including *Fusobacterium* spp., *P. gingivalis*, and *T. forsythia* were detected in significantly higher quantities in the obese patients (diabetics and nondiabetics) than nonobese nondiabetic control. Resistin concentrations were significantly correlated with BMI; however, its level was not correlated with the blood glucose. In this study, high salivary resistin was associated with obesity, which is a major predisposing factor for type 2 diabetes and also a risk factor for oral diseases. The high levels of salivary periodontopathogenic bacteria could upregulate the local release of salivary resistin in obese people.

## 1. Introduction

Obesity is a major public health problem today. It is a risk factor for several chronic diseases, most notably hypertension, dyslipidemia, coronary heart diseases, and diabetes. Diabetes is a chronic metabolic disorder that occurs due to insufficient pancreatic secretion of insulin (type 1 diabetes) or due to the inefficient use of the insulin by the body cells (type 2 diabetes) [1]. According to the International Diabetes Federation statistics in 2015, 1/11 adults (415 million) have diabetes and it is expected that 1/10 adults (642 million) will have diabetes in 2040 [2].

A biomarker, or biological marker, generally refers to a measurable indicator of physiologic health, a pathogenic process, or a pharmacologic response to a therapeutic intervention. Whether a biomarker is produced by healthy individuals or by individuals affected by a particular systemic disease, these molecules can be used to monitor health status, disease onset, treatment response, and outcome [3]. Human saliva is an ultrafiltrate from plasma; thus, it can be used as an alternative to serum for the detection of diagnostic biomarkers, such as resistin [4]. Resistin (resist insulin) is an adipocytokine, produced by adipocytes and macrophages. In mice, resistin was proposed as a possible link between obesity and insulin resistance; however, its exact role in the pathogenesis of type 2 diabetes in human is still a matter of debate. Previous studies reported a positive correlation between serum and salivary resistin, which were both correlated with BMI in type 2 diabetes patients [4]. Data suggests that resistin could be one of the molecular links connecting obesity, diabetes, and periodontitis and may serve as a link between periodontal diseases and other systemic diseases [5].

Obesity is one of the factors that predispose to periodontal diseases. Limited studies have described the oral microbiology of obese individuals. Periodontal diseases usually range from the mild reversible form of infection (gingivitis) to the more severe and irreversible form of the disease (periodontitis) which involves destruction of the alveolar bone supporting the tooth and eventually leads to tooth loss. Periodontopathogenic bacteria particularly the red complex group (*Treponema denticola*, *Porphyromonas gingivalis*, and *Tannerella forsythia*) are the main causative agents of periodontal diseases [6]. Traditional culture techniques used to study the oral bacteria are time-consuming, with limited low sensitivity, expensive, and unable to detect oral bacteria which are difficult to culture or nonculturable. Additionally, only viable bacteria can grow on culture; therefore, strict sampling and transport conditions are essential [7]. The use of molecular techniques facilitates the characterization of both cultivable and noncultivable members of the oral bacteria. Recent reports indicate that up to 1000 phylotypes could potentially colonize the oral cavity [8].

This study aims to investigate the relation between resistin and the levels of periodontopathogenic bacteria in the saliva of obese adults (diabetics and nondiabetics) compared to a nonobese nondiabetic control.

## 2. Materials and Methods

A cross-sectional study was conducted in the period between December 2015 and April 2016. In this study, 78 adult patients attending to the University of Sharjah Dental Hospital, Sharjah, UAE, were recruited. After signing the consent form, the participants were interviewed to take their medical history. Dental examination was done by the same dentist as part of the routine screening procedure. Oral examination was performed to ensure the absence of severe periodontal destruction using the clinical periodontal sum score (CPSS) which is the sum of the number of sites with probing pocket depths of ≥4 mm, the number of gingival sites with bleeding after probing, the visible suppuration, and the number of furcation lesions exceeding grade 1 [9]. Fasting blood glucose was measured for each patient. Weight and height were measured using a standard scale and a fixed height bar. BMI was calculated by dividing the weight (kg) by the squared height (m^2^).

### 2.1. Inclusion Criteria

The inclusion criteria were as follows:

- Age between 18 and 60
- Belonging to one of the following groups (*n* = 26 in each group):
  1. obese diabetics,
  2. obese nondiabetics,
  3. nonobese nondiabetics.

Obese group included individuals with BMI ≥ 30, whereas nonobese group included those with BMI < 30. Presence or absence of diabetes was based on self-reporting by the patients. Only patients with type 2 diabetes (adult onset, non-insulin-dependent diabetes) were included in the study.

### 2.2. Exclusion Criteria

The exclusion criteria were as follows:

- Having other endocrine diseases
- Having other systemic diseases
- Having chronic inflammatory diseases
- Patients currently taking antibiotics or anti-inflammatory agents and those who took them over the past month
- Pregnant ladies
- Patients with severe periodontal destruction: those having at least four sites with probing pocket depth (PPD ≥ 4 mm) and clinical attachment level (CAL ≥ 1 mm) [10].

Patients with the abovementioned conditions were excluded as they may affect the detected resistin concentration [5, 11].

### 2.3. Saliva Sample Collection

A total of 78 saliva samples were collected from the patients. Saliva collection was done between 9.00 and 10.00 am in a separate dental clinic setup for this purpose. The patients were asked to refrain from drinking, eating, and tooth brushing one hour prior to sampling. The mouth was rinsed with water to remove any food residue, and the sample was collected after 10 minutes. Unstimulated whole saliva was collected from each patient by passive drool method over 5 minutes. Briefly, the participants sat at a comfortable resting upright position with their heads tilted down slightly to pool saliva in the mouth [12]. Saliva was then spitted into a sterile labelled container. The collected saliva was placed in an ice container, then transferred immediately to −20°C freezer until testing within one month after sampling.

### 2.4. Determination of Resistin Concentration

Resistin concentration in each saliva sample was determined using a Resistin Human ELISA Kit (ab108896, Abcam, UK). Briefly, 2 ml of each saliva sample was centrifuged at 800*g* for 10 minutes; then, the supernatant was used for the assay according to the manufacturer's instructions. The absorbance was measured on a microplate reader at a wavelength of 450 nm. Absorbance values were used to calculate the concentrations using a standard curve by four-parameter logistic analysis. For confirmation, resistin concentration was tested in duplicate for some selected cases.

### 2.5. Bacterial DNA Extraction from the Saliva

Bacterial DNA was extracted from saliva samples using a commercial DNA extraction kit (Norgen, Canada) according to the manufacturer's recommendations. Briefly, the cell pellet generated from the centrifugation of 2 ml saliva sample was suspended in 500 *μ*l of sterile phosphate buffer saline and boiled for 10 minutes. The samples were then cooled on ice for two minutes and centrifuged at 14,000 rpm for 5 minutes. The supernatant was then mixed with 300 *μ*l of the lysis buffer provided in the kit. Then, 600 *μ*l of the resultant solution was transferred into the spin column provided in the kit and centrifuged at 8000 rpm for one minute. Spin column washing was done twice using 500 *μ*l of the wash buffer provided in the kit and followed by centrifugation at 6000 rpm for one minute. After centrifugation at 14,000 rpm for 2 minutes for complete drying of the column, DNA elution was done by loading the column with 20 *μ*l of the elusion buffer provided in the kit. DNA was collected into a sterile microcentrifuge tube by 3 times centrifugation: first at 2000 rpm for one minute, followed by 8000 rpm for two minutes, and finally at 14,000 rpm for 30 seconds. The resultant DNA was stored at −20°C until use in PCR.

### 2.6. Bacterial Quantification by Real-Time PCR

Real-time PCR was carried out with a Rotor-Gene® Q PCR thermocycler (Qiagen, Germany). Amplification was performed in a total reaction volume of 20 *μ*l containing 5x HOT FIREPol® EvaGreen® qPCR Mix Plus (Solis BioDyne, Estonia) and optimized concentration (0.2 *μ*M) of primers manufactured by Macrogen, Korea. The primers used in real-time PCR with the annealing temperatures for each assay are listed in Table 1. All the PCR amplification reactions were performed in duplicate.

### 2.7. Real-Time PCR Analysis

The critical threshold cycle (Ct) is defined as the first cycle in which fluorescence is detectable above the background and is inversely proportional to the logarithm of the initial number of template molecules. The fold difference (*N*) in the number of the target organism-specific gene copies relative to the number of 16S rRNA gene copies was determined as follows:

(1) $$\begin{array}{c} N=2^{-\Delta Ct}. \end{array}$$

The ∆Ct for each sample was determined by subtracting the Ct value of the target gene from that of the 16S rRNA gene (universal primers) of the same sample, that is, ∆Ct = Ct target DNA − Ct 16S rRNA [19].

### 2.8. Statistical Analysis

The statistical tests were performed using SPSS Statistics version 21 (SPSS Inc., Chicago, IL, USA). Results were expressed as mean ± standard deviation. A *p* value <0.05 was considered statistically significant. Continuous variables were compared by Mann–Whitney *U* or Kruskal-Wallis tests as appropriate. Correlation was tested using Spearman's correlation coefficient.

## 3. Results

The general information of the study population and laboratory results is summarized in Table 2. The full list of the cases and their laboratory results is provided in the supplementary data file available online at https://doi.org/10.1155/2017/2643079.

As shown in Table 2, there was significant difference in the weight, BMI, blood glucose, and salivary resistin between obese patients (diabetics and nondiabetics) and the nonobese nondiabetic control. The difference between diabetic and nondiabetic obese patients was not significant for all the tested variables (*p* values >0.05), except for blood glucose levels (*p* value <0.001). Salivary resistin was significantly higher in the obese patients (diabetics and nondiabetics) compared to the nonobese nondiabetic control. There was a significant correlation between salivary resistin levels and BMI (*p* value: 0.007). BMI was significantly correlated not only with salivary resistin levels but also with blood glucose and weight (*p* value <0.001). There was no correlation between salivary resistin levels and blood glucose (*p* value: 0.051).

Quantification of oral bacteria using real-time PCR revealed significantly higher quantities of *Fusobacterium* spp. (associated with gingivitis), *P. gingivalis*, and *T. forsythia* (associated with periodontitis) in the obese patients (diabetics and nondiabetics) compared to the nonobese nondiabetic control, as shown in Table 3. The difference between diabetic and nondiabetic obese patients in the levels of all the tested bacteria was not significant.

Regarding the prevalence of the bacteria in the saliva samples, *Fusobacterium* spp. was the most prevalent bacterial type in this study (*n* = 78, 100%), followed by *P. gingivalis* and *T. forsythia* (*n* = 76, 97.4% each). *T. denticola* was detected in 69 samples (88.5%), whereas *A. actinomycetemcomitans* was detected in 24 samples (30.8%) only. The quantities of *Fusobacterium* spp., *P. gingivalis*, *T. forsythia*, and *A. actinomycetemcomitans* were significantly correlated with BMI (*p* values: 0.003, <0.001, <0.001, and 0.015) for each bacteria, respectively.

There was no significant difference between obese (diabetics and nondiabetics) and nonobese groups with respect to the total number of bacteria and periodontal health condition (*p* values: 0.185 and 0.164, resp.), as shown in Table 4.

There was no correlation between the levels of salivary resistin with the total number of oral bacteria and with the quantity of different oral bacteria in the saliva samples.

## 4. Discussion

Nowadays, obesity is considered a chronic inflammatory condition [20]. In obese individuals, the inflamed adipose tissue acts as a gland for the secretion of several hormones including resistin. Data suggests that resistin can target multiple cell types, including inflammatory cells, vascular endothelial and smooth muscle cells, cardiomyocytes, and hepatocytes [21]. Resistin can cause malfunction of these cells; therefore, it is involved in the pathogenesis of many obesity-related disorders including cardiovascular diseases such as atherosclerosis and coronary heart diseases, type 2 diabetes mellitus, and fatty liver, [21].

In this study, there was a significant correlation between the levels of salivary resistin and BMI, as reported previously [4]. No correlation was found between salivary resistin levels and blood glucose; however, BMI was significantly correlated with blood glucose. This study reflects that the increase in resistin is associated with obesity, which is a predisposing factor for type 2 diabetes mellitus [22]. The correlation between obesity and resistin was supported by the findings of studies concerning obesity management, whereby weight loss by diet and exercise or bariatric surgery led to a reduction in resistin in parallel with BMI reduction [23, 24]. Animal studies suggest that the role of resistin in the pathophysiology of type 2 diabetes mellitus is through inflammation-induced insulin resistance [21]. In one study, diet-induced obesity was investigated in humanized resistin mice, which are transgenic mice producing resistin mainly from macrophages in a way similar to human. Inflammation of the white adipose tissue and subsequent insulin resistance were observed in these mice [25].

In this study, both salivary resistin and certain oral bacterial species (*Fusobacterium* spp., *P. gingivalis*, and *T. forsythia*) were detected in significantly higher quantities in the obese patients (diabetics and nondiabetics) compared to the nonobese nondiabetic control. The levels of resistin detected in the saliva of the obese patients (diabetics and nondiabetics) were significantly higher than those of the nonobese nondiabetic control. The level of resistin detected in the saliva is high (14.7 ± 2.8 and 14.4 ± 3.6 ng/ml in the diabetics and nondiabetic obese patients, resp.) compared to the level reported by Yin et al. in 2012, whereby salivary resistin was 3.4 ± 0.4 ng/ml in diabetic patients. The high levels reported in our study might be attributed to the local release of resistin by the macrophages in the tissue of the oral cavity [5, 26] or secretion by the adipocytes of the salivary glands [5, 10]. The bacteria present in high quantity in the oral cavity of obese patients (diabetics and nondiabetics) might be the trigger of this local release through the effect of lipopolysaccharides (LPS). LPS (also known as endotoxins) are part of the cell wall of Gram-negative bacteria that has been demonstrated to induce inflammatory reaction as well as the expression of resistin. Previous *in vitro* studies reported the release of resistin from human neutrophils after the addition of LPS derived from periodontopathogenic bacteria, in particular *P. gingivalis* [27, 28] and *A. actinomycetemcomitans* [29]. Additionally, previous animal studies reported that the expression of resistin by adipocytes and white blood cells was upregulated after the administration of LPS in rats [30]. A previous study reported a dose-dependent increase in resistin gene and protein expression by the human macrophages and by healthy human subjects with induced endotoxemia following LPS administration [31]. The latter study reported that LPS induced the production of proinflammatory mediators (such as TNF-*α* and IL-6), which in turn induced resistin expression [31]. On the other hand, other studies reported that resistin itself upregulated the expression of proinflammatory cytokines from peripheral blood mononuclear cells via the nuclear factor-*κ*B (NF-*κ*B) pathway [5, 21]. Thus, the relation between inflammation and resistin expression seems to be bidirectional, associated with the release of several proinflammatory cytokines which are involved in tissue injury [11]. Resistin has been suggested as a potential link between inflammation and metabolic diseases as the inflammatory nature of many metabolic disorders including diabetes has been shown in many clinical and basic research studies [11]. Many clinical reports documented the detection of high levels of resistin in patients with various chronic inflammatory conditions such as rheumatoid arthritis, inflammatory bowel diseases, and chronic kidney diseases and those with oral disease, particularly periodontitis [5, 21, 31].

Many studies reported the detection of higher levels of resistin in the serum [26], gingival crevicular fluid [27], or saliva [10] of patients with chronic periodontitis. Studying the oral microbes in relation to resistin was overlooked in all the previous clinical studies. A recent clinical study reported high levels of salivary resistin (14.45 ± 1.88 ng/ml) in normal-weight patients with chronic periodontitis compared to those with gingivitis (11.59 ± 1.6 ng/ml) and periodontally healthy cases (6.43 ± 0.81 ng/ml) [10]. Although most of our cases were periodontally healthy, salivary resistin levels in our obese cases whether diabetics (14.7 ± 2.8 ng/ml) or nondiabetics (14.4 ± 3.6 ng/ml) were close to the levels reported by Karam and Al-Safi in normal-weight patients with chronic periodontitis (14.45 ± 1.88 ng/ml). Karam and Al-Safi did not explore the periodontopathogenic bacteria in the saliva of the investigated cases; however, they assumed higher microbial content in the oral cavity of the cases with chronic periodontitis as a trigger for the local release of high levels of salivary resistin.

The oral cavity is inhabited by three types of microbes: symbionts (have health-promoting functions), commensals (with no benefit or harm to the host), and pathobionts (can induce pathology under certain conditions). These microbes live in symbiotic relationship without causing any harm to the host. When the normal balance or homeostasis between these microbes is lost (dysbiosis), disease conditions like periodontitis evolved [32]. This homeostasis is affected by the immune condition of the host; thus, in patients with systemic metabolic disease like diabetes, the risk for developing periodontal disease is high [33]. There are several consecutive steps in the pathogenesis of periodontitis starting from the acquisition of the bridging bacteria (e.g., *Fusobacterium*) which provide a scaffold for linking the keystone pathogens like *P. gingivalis*, *T. forsythia*, and *T. denticola*. These pathogens work in a collaborative manner leading to the stimulation of the inflammatory cells in the periodontal tissue. This inflammatory response leads to a cascade of events with secretion of several cytokines involved in the process of destruction of the alveolar bone supporting the tooth [34]. In this study, we report the detection of the bridging bacteria (*Fusobacterium* spp.) and keystone pathogens (*P. gingivalis* and *T. forsythia*) in significantly higher quantities in the obese patients (diabetics and nondiabetics) compared to the nonobese nondiabetic control. Furthermore, the quantities of these bacteria were significantly correlated with BMI. Although the obese patients in our study were periodontally healthy (Table 4), they are possibly at the preclinical stage of the disease and they may be at risk of developing periodontal disease in the future if they did not take good care of their oral health. Several studies have demonstrated lower prevalence of periodontitis, lower levels of inflammatory markers, and higher insulin sensitivity in normal-weight individuals, especially those engaged in physical activities [35, 36]. Additionally, it was noted that the response to nonsurgical periodontal therapy was better in obese patients after significant weight loss compared to those who did not lose any weight [37].

Oral health is linked to general health; thus, the presence of significantly higher quantities of bacteria in the saliva of obese patients may put them at higher risk of developing systemic complications. As 1–1.5 litres of the saliva are swallowed per day, the bacteria in the saliva can interact with gastrointestinal flora altering the composition of the gut microbiota [33]. Additionally, the bacterium *P. gingivalis* has been proven to alter the epithelial lining of the gut, making it more permeable, and then releasing its endotoxin (LPS) which can enter the systemic circulation leading to systemic inflammation [33]. It has been also reported that systemic endotoxemia caused insulin resistance in healthy human volunteers after LPS administration [38].

As a template of PCR, saliva has been reported to be superior to the dental plaque samples because it is more representative for the overall oral health condition and due to the higher chance for recovering the bacteria causing periodontitis and dental caries [39]. Detection of microbes in the saliva using quantitative real-time PCR can be used in the diagnosis of the microbial causes of oral infectious diseases, especially for unculturable oral bacteria. It is also important to predict the prognosis, that is, monitoring the effect of therapy and predicting the risk of recurrence.

## 5. Conclusions

In this study, salivary resistin was correlated with obesity which is a predisposing factor for type 2 diabetes. It was difficult to conclude that resistin is a biomarker for diabetes as there are several factors affecting its level, like the inflammatory status of the subjects, including the oral health condition which was not considered in many of the previous studies linking resistin to diabetes [5].

In this study, multiple important periodontopathogenic bacteria were detected in the saliva of obese individuals. The high resistin level in obese people may be linked to the microbiota inhabiting the mouth as LPS from periodontopathogenic bacteria has been reported as an inducer for the expression of resistin [27–29]. Our study depended on noninvasive saliva sampling without invasive blood collection [40]. Further studies must be conducted in the future to compare the levels of resistin in the saliva and serum in both periodontally healthy and diseased patients in order to confirm the role of the periodontopathogenic bacteria in the induction of local resistin secretion in the saliva.

Due to the high oral microbial contents, obese people are probably at risk for developing invasive periodontal diseases, unless good oral health interventions are adopted. Many patients do not realize that they have periodontal diseases as they are silent and asymptomatic in nature. Meanwhile, physicians may not know that the patient has an oral disease that affects sugar control and complicates diabetes management [41]. The relationship between oral and general health will challenge dentists and physicians to work together in managing patients with periodontal diseases and systemic diseases like diabetes. Therefore, patients with diabetes should consult a dentist for periodontal screening, and patients with periodontal disease should be screened for diabetes if signs or symptoms are present. Dentists should explain to the obese individuals about the possible oral complications of obesity and should follow up their oral condition in order to diminish morbidity associated with obesity.

## Supplementary Material

List of the investigated cases (non-obese and non-diabetics, obese non-diabetics & obese diabetics) with their laboratory results.

## Figures and Tables

**Table 1:** List of the primers used in real-time PCR experiments with the annealing temperatures for each assay.

Bacterial species (target gene)	Primer sequence (5′–3′)	Annealing temperature (°C)	Reference
*Actinobacillus actinomycetemcomitans* (iktA)	CAG CAT CTG CGA TCC CTG TA	58	[13]
	TCA GCC CTT TGT CTT TCC TAG GT		
*Porphyromonas gingivalis* (16S rRNA)	ACC TTA CCC GGG ATT GAA ATG	58	[14]
	CAA CCA TGC AGC ACC TAC ATA GAA		
*Treponema denticola* (16S rRNA)	AGA GCA AGC TCT CCC TTA CCG T	58	[15]
	TAA GGG CGG CTT GAA ATA ATG A		
*Tannerella forsythia* (16S rRNA)	GGG TGA GTA ACG CGT ATG TAA CCT	55.5	[16]
	ACC CAT CCG CAA CCA ATA AA		
*Fusobacterium* spp. (16S rRNA)	CGC AGA AGG TGA AAG TCC TGT AT	58	[17]
	TGG TCC TCA CTG ATT CAC ACA GA		
Universal primers (16S rRNA)	TCC TAC GGG AGG CAG CAG T	60	[18]
	GGA CTA CCA GGG TAT CTA ATC CTG TT		

**Table 2:** General information of the study population and laboratory results. The figures shown are mean ± standard deviation.

Parameters	Obese		Control (nonobese and nondiabetics) (*n* = 26)	*p* value
	Diabetics (*n* = 26)	Nondiabetics (*n* = 26)		
Age (years)	51.1 ± 5.7	47.9 ± 5.7	47.4 ± 5	0.055
Body mass index (kg/m^2^)	34.3 ± 3.9	34.2 ± 2.9	27.1 ± 2.1	<0.001^∗^
Weight (kg)	94.5 ± 12.4	97.3 ± 13.3	77.5 ± 12.1	<0.001^∗^
Height (cm)	166 ± 10.4	168.5 ± 8.5	168.7 ± 10.2	0.492
Fasting blood glucose (mg/dl)	205.5 ± 83.9	106.2 ± 24.9	94 ± 17.1	<0.001^∗^
Resistin (ng/ml)	14.7 ± 2.8	14.4 ± 3.6	10.8 ± 6.1	0.010^∗^

^∗^Significant difference (*p* < 0.05) between obese and nonobese groups.

**Table 3:** Real-time PCR quantification of *Fusobacterium* spp., *P. gingivalis*, *T. forsythia*, *T. denticola*, and *A. actinomycetemcomitans* in the saliva of obese patients (diabetics and nondiabetics) compared to the nonobese nondiabetic control.

Bacterial species	*N* range (mean ± SD) for each group			*p* value
	Nonobese (*n* = 26)	Obese diabetics (*n* = 26)	Obese nondiabetics (*n* = 26)	
*Fusobacterium* spp.	3 × 10^−5^–6.9 × 10^−2^	12 × 10^−5^–100.7 × 10^−2^	60 × 10^−5^–80.7 × 10^−2^	0.003^∗^
	(1.1 ± 1.6 × 10^−2^)	(11.5 ± 22.5 × 10^−2^)	(10.1 ± 20.5 × 10^−2^)	
Positive cases per group	26	26	26	Total: 78 (100%)

*P. gingivalis*	0.45 × 10^−5^–0.7 × 10^−2^	1.87 × 10^−5^–13.49 × 10^−2^	0.06 × 10^−5^–46 × 10^−2^	<0.001^∗^
	(0.08 ± 0.16 × 10^−2^)	(1.6 ± 3.4 × 10^−2^)	(5.2 ± 11.6 × 10^−2^)	
Positive cases per group	26	26	24	Total: 76 (97.4%)

*T. forsythia*	0.5 × 10^−5^–0.14 × 10^−2^	3.7 × 10^−5^–2.3 × 10^−2^	17.1 × 10^−5^–14.46 × 10^−2^	<0.001^∗^
	(0.04 ± 0.04 × 10^−2^)	(0.73 ± 0.74 × 10^−2^)	(1.9 ± 3.2 × 10^−2^)	
Positive cases per group	24	26	26	Total: 76 (97.4%)

*T. denticola*	0.2 × 10^−5^–0.34 × 10^−3^	0.1 × 10^−5^–4.6 × 10^−3^	0.2 × 10^−5^–8.2 × 10^−3^	0.068
	(8.9 ± 9.9 × 10^−5^)	(37.99 ± 92.8 × 10^−5^)	(58.9 ± 168.2 × 10^−5^)	
Positive cases per group	20	25	24	Total: 69 (88.5%)

*A. actinomycetemcomitans*	0.3 × 10^−4^–2.1 × 10^−4^	0.2 × 10^−4^–4.9 × 10^−3^	6.8 × 10^−4^–24.5 × 10^−3^	0.097
	(0.5 ± 0.8 × 10^−4^)	(0.9 ± 1.7 × 10^−4^)	(5.7 ± 8.9 × 10^−4^)	
Positive cases per group	7	8	9	Total: 24 (30.8%)

*N* = fold difference in the number of the target organism-specific gene copies relative to the number of 16S rRNA gene copies. ^∗^Significant difference (*p* < 0.05).

**Table 4:** Total number of bacteria in the saliva of obese patients (diabetics and nondiabetics) compared to the nonobese nondiabetic control.

Periodontal condition	Number of cases per group (number of bacteria)		
	Nonobese nondiabetics (*n* = 26)	Obese diabetics (*n* = 26)	Obese nondiabetics (*n* = 26)
Healthy (*n* = 67)	20 (3–6)	25 (4–6)	22 (3–6)
Nonhealthy^∗^ (*n* = 11)	6 (3–6)	1 (6)	4 (4–6)
Total number of bacteria	3–6	4–6	3–6

^∗^Nonhealthy: those with mild form of periodontal diseases, that is, gingivitis.
